# Curious Case of Periodical Vino-Mania

**Published:** 1829-01-01

**Authors:** 


### PERIODICAL VINO-MANIA.

If the "March of Intellect," the Catholic Rent, or the thirst for political freedom, has not refined the tastes of the Hibernian peasantry, there may be occasionally seen among them a periodical disease, which has not yet found a place in any system of nosology. We shall call it the WHISKEY-FEVER, on the authority of Dr. Bostock, who designates one of our English annuals, the HAY-FEVER. The disease, or rather *disorder* to which we allude, is not habitual intemperance, but an ardent paroxysm of whiskey-drinking, lasting from two or three days to as many weeks; in which, drinking and sleeping, enlivened by an occasional song or a fight, constitute the whole of the phenomena during the paroxysm. This over, the patient comes out of his fever with unenviable feelings of mental remorse and corporeal misery, (not much alleviated by the reproaches of his wife or connexions) during which period, he generally takes an oath not to touch the CREATURE for a year and a day. The oath is strictly kept; but the 366th day is scarcely closed, when a paroxysm of the whiskey-fever makes its approach in the shape of a hearty pre-determination to—

> "Tak a right gude willy-waugh,
> "For Auld Lang Syne."

It is probable, however, that, were Goldsmith to revisit his Native Village now, he would have to deplore the decadence of another of those good old customs which annually enlivened the heart of the rustic peasant.

"These were his joys, but all these joys are fled."

The whiskey-fever of the Irish peasant was brought to our recollection by the narrative of a case of periodical Vino-mania, in the JOURNAL DE PROGRÉS, and to which is given the appellation "IVRESSE INVOLONTAIRE PERIODIQUE." The individual was a very eminent bookbinder, of Madrid, who, in consequence of some domestic chagrins, quitted the capital, and settled in Valentia. There he got married again, and carried on his business with much reputation, during the intervals of his malady. This disorder commenced fifteen years ago, in the shape of an irresistible impulse to swallow wine, day and night, without the possibility of satiety. The paroxysm lasts, usually, from two to three months, with an interval of equal duration, when it returns again, without any prodrome that might intimate its approach. In the intervals, he seems to recollect nothing of the paroxysm—never speaks of the transactions, and either will not, or cannot give any information respecting his feelings on these occasions. The narrator, M. Pierquin, who has watched him for some years, has noted the following particulars. When the vino-mania occurs, the man gets up at five or six o'clock in the morning, rifles the TILL of the counter of its contents, repairs to the nearest tavern, and there ingurgitates wine at a tremendous rate, till 10 or 11 o'clock, when he staggers home, goes down to his cellar, brings up some large bottles of wine, and drinks day and night —seldom sleeping, and very rarely eating any food. During the early period of the attack, he usually goes out now and then to the cabaret; but during the last 18 or 20 days of the fit, he never stirs from home. He is then taciturn, choleric, and scolds his wife — avoids the light, and skulks into the darkest recesses of the kitchen, where he drinks and grumbles from morning till night. Notwithstanding his ebullitions of temper, he is never delirious or deranged in his intellects, on these occasions; but answers correctly, and follows the chain of a discourse, when he can be brought into conversation. The paroxysm ends in a profound sleep, from which he awakes in his sober senses, and resumes his avocations as if he had just quitted them the preceding evening—apparently unconscious of any thing that had passed.

The above is, of course, a very different affection from the periodical WHISKEY-FEVER of honest Pat. It bears some analogy to somnambulism, and though the narrator avers that there is no proof of mental aberration present in the paroxysm, yet we cannot help viewing it as one of the anomalous forms of mental derangement. The narrator informs us, that he has lately seen the individual alluded to. The complaint has now lasted upwards of 15 years, with little alteration in itself, though the sufferer is melancholy, fond of solitude, evincing an increasing degree of hebetude.—*Journal de Progrés.*

